# The activated newborn neurons participate in enriched environment induced improvement of locomotor function in APP/PS1 mice

**DOI:** 10.1002/brb3.1316

**Published:** 2019-05-15

**Authors:** Hualong Wang, Qiongqiong Li, Huidong Tang, Jianqing Ding, Nanjie Xu, Suya Sun, Shengdi Chen

**Affiliations:** ^1^ Department of Neurology Ruijin Hospital, Shanghai Jiao Tong University School of Medicine Shanghai China; ^2^ Department of Anatomy and Physiology Shanghai Jiao Tong University School of Medicine Shanghai China

**Keywords:** Alzheimer's disease, elevated plus maze, enriched environment, locomotor function, neurogenesis

## Abstract

**Introduction:**

Alzheimer's disease (AD) is an age‐related neurodegenerative disorder. One of the pathological features of AD is neuronal loss in brain regions associated with cognition, particularly the hippocampus. An enriched environment (EE) can facilitate neuronal plasticity and improve behaviors such as emotion, motor function, and cognition in AD.

**Methods:**

After APP/PS1 mice were exposed to EE at an early stage (2 months of age), elevated plus maze performance and contextual fear conditioning were tested, and neurogenesis and the extent of activation in the hippocampus were observed.

**Results:**

The results showed that, compared with that in the mice that experienced a standard environment, the cognition of the mice exposed to EE, as measured by contextual fear conditioning, was not statistically significant. However, based on their performance in the elevated plus maze, the index was increased in the mice, especially the APP/PS1 mice, exposed to EE. Consistent with the behavioral changes, the APP/PS1 mice exposed to EE showed an increased number of c‐Fos‐positive neurons and elevated neurogenesis in the dentate gyrus (DG) area. In addition, the activation of newborn neurons did not occur in the other three groups.

**Conclusions:**

These results indicate that the activation of newborn neurons may participate in the improvement of behavioral performance in APP/PS1 mice after EE.

## 1 INTRODUCTION

One of the pathological features of Alzheimer's disease (AD), an age‐related neurodegenerative disorder, is neuronal loss in brain regions associated with cognition, particularly the hippocampus. Neurogenesis occurs in two major brain regions: the subgranular zone in the dentate gyrus (DG) of the hippocampus and the subventricular zone of the lateral ventricles (Ming & Song, 2011). The hippocampus is involved in learning and memory, emotion and locomotion, and neurogenesis in the hippocampus is quite vital for normal behaviors (Deng, Aimone, & Gage, 2010). A large number of studies have shown that the impairment of hippocampal neurogenesis and neuronal excitability may be closely correlated with hippocampal dysfunction in AD, and an increase in neurogenesis and/or a decrease in neuronal excitability can improve cognitive behaviors in animal models (Becker, Lavie, & Solomon, 2007; Clelland et al., 2009; Lazarov, Mattson, Peterson, Pimplikar, & van Praag, 2010; Sahay et al., 2011; Tchantchou, Xu, Wu, Christen, & Luo, 2007; Wang et al., 2017). After the process of neurogenesis, these newborn neurons become functionally active and are thought to contribute to the normal functioning of the hippocampus (Goncalves, Schafer, & Gage, 2016).

An enriched environment (EE) comprises a variety of social and physical stimuli, such as objects of various shapes and sizes in different locations. Compared with standard environment (SE) conditions, EE can improve behaviors related to altered neuronal plasticity, such as emotion, motor functions, learning and memory, in normal rodents, as well as in models of neurodegenerative diseases, such as AD (Lee & Trojanowski, 2006). Numerous studies have indicated that EE plays an important role in facilitating dendritic growth, inducing hippocampal neurogenesis (Catlow et al., 2009; Holloway, 1966; Hu et al., 2010), and improving cognitive and motor functions in various behavioral tasks (Cotel, Jawhar, Christensen, Bayer, & Wirths, 2012; Frick & Fernandez, 2003; Goshen et al., 2009). c‐Fos is a proto‐oncogene that is expressed in some neurons following depolarization. The protein product, the c‐Fos protein, can be identified by immunohistochemical techniques. Therefore, c‐Fos expression can be used as a marker of neuronal activity throughout the neuraxis following peripheral stimulation (Bullitt, 1990).

Previous studies have shown that behavioral, psychological, and personality symptoms emerge in the early stage of AD (von Gunten, Pocnet, & Rossier, 2009; Pocnet, Rossier, Antonietti, & von Gunten, 2013). Our preliminary results showed that APP/PS1 mice exhibit emotional dysfunction at 2 months. However, cognitive performance is not changed compared with that of wild‐type mice at this age, even though it declines during aging (Pentkowski et al., 2018). Therefore, we speculated that abnormal emotion and behavior performance, which may play a key role in the pathogenesis of AD, emerges in the early stage of dementia.

The present study aimed to elucidate whether EE is effective in an early stage in APP/PS1 mice. We found that the APP/PS1 mice exposed to SE conditions exhibited impairments in hippocampal neurogenesis and worse locomotion function, while in those exposed to EE, these two measures were improved upon the activation of newborn neurons.

## 2 MATERIALS AND METHODS

### 2.1 Animals, EE, and EdU administration

APP/PS1 mice (Thy1–APPKM670/671Nl, Thy1–PS1 L166P) were kindly provided by Mathias Jucker (Tubingen University). The generation and characterization of APP/PS1 mice has been described previously (Pentkowski et al., 2018). At the age of three weeks, 16 male wild‐type mice on a 129/Sv background and 18 male APP/PS1 mice were intraperitoneally injected with 100 mg/kg of EdU once a day for 7 days (ThermoFisher Scientific) and were then randomly assigned to either SE or EE housing conditions for 1 month. Standard laboratory cages (33 cm × 18 cm × 14 cm) were used for SE, whereas larger cages (55 cm × 34 cm × 20 cm) with various toys were used for the EE condition (Hu et al., 2010). In the EE cages, the animals also had access to a variety of different objects, such as running wheels, colored tunnels, and visually stimulating toys, which were changed weekly. All animals were tested on the elevated plus maze (EPM) and a fear conditioning task after 1 month of experience with SE or EE.

All mice were housed in conditions of constant temperature (20–22°C) and a 12‐hr light–dark cycle with access to food and water ad libitum. All animal experiments were performed under an animal study protocol approved by the ethics committee of Shanghai Jiao Tong University School of Medicine.

### 2.2 EPM

All tests were conducted according to a previous study (Zhu et al., 2016). After EE or SE exposure, the mice were habituated to handling and were transported from the colony room to the behavioral room for 3 days before behavioral tests were performed. The mice were given 1 hr to habituate after transport to the behavioral room before any tests were conducted. All apparatuses and testing chambers were cleaned with 75% ethyl alcohol wipes between animals. The EPM apparatus was made of dark gray plastic and consisted of two open arms (30 × 7 × 0.25 cm) opposing two enclosed arms (30 × 7 × 15 cm) elevated 60 cm from the floor. The animals were placed in the central area of the apparatus with their head facing an enclosed arm (test duration: 5 min). The test was performed in a sound‐attenuated and temperature‐controlled (22 ± 1°C) room illuminated by one 40‐W fluorescent bulb placed 3 m above the apparatus. Digitized video recordings (30 frames per s) obtained by EthoVision software (Noldus Information Technology, Leesburg, VA) were used for behavioral analysis. The time spent in the open arms and the frequency of entry into the open arms were used as innate behavioral indexes.

### 2.3 Contextual fear conditioning

The contextual fear conditioning test was performed using a UGO Basile Fear Conditioning System (Model 46850). During the training session, each mouse was placed in a shock chamber for 3 min. Black‐and‐white striped wallpaper applied to the sides of the experimental cage, tactile sensations on the feet of the animals, and the odor of alcohol formed the contextual information for context A of fear conditioning training. During the training session, the mice were first acclimated to the experimental cage for 3 min and then exposed to by a paired voice (4 kHz, 76 db, duration of 30 s) and plantar electric shock (0.85 mA, 2 s). After the plantar electric shock, the mice stayed in the experimental cage for an additional 30 s. At the end of the experiment, the mice were returned to the breeding cage. After each mouse experiment, the test chamber was wiped with 75% ethanol. Twenty‐four hours later, the animals were subjected to fear conditioning test A (fear A). First, each mouse was placed in the context A environment, and no sound stimulation was given. The recording was performed for 3 min. After each mouse experiment, the test chamber was wiped with 75% ethanol. Three hours later, the experimental mice were then subjected to fear conditioning test B (fear B). The sides of the chamber were covered with light gray wallpaper, the bottom was covered with a smooth gray plastic plate, and the experimental cage was wiped with 4% acetic acid solution instead of 75% ethanol (context B). During the experiment, the animals first adapted to the new environment for 3 min, and then received a plantar electric shock once. Recordings continued for 2 min after the plantar electric shock. After each mouse was tested, the chamber was wiped with 4% acetic acid. The freezing times in the fear A and fear B tests were recorded as the cognitive index.

### 2.4 Immunofluorescence

For immunofluorescence, coronal brain slices were sectioned at a thickness of 30 μm, washed with phosphate buffered saline (PBS), and then incubated for blocking with permeabilization buffer (0.3% Triton‐100 in PBS) containing 10% donkey serum for 45–60 min. The sections were incubated in primary antibodies against NeuN (Millipore) and c‐Fos (Cell Signaling Technology) overnight at 4°C. The sections were rinsed three times (10 min each) in PBS, permeabilized with 0.1% Tween‐20 in PBS, and then incubated at 4°C overnight with Alexa Fluor secondary antibodies specific for the primary antibody host. Following incubation with secondary antibodies, the sections were rinsed three times (10 min each) in PBS, mounted on gelatin‐coated glass slides, and coverslipped using mounting medium. For EdU staining, we used Click‐iT^®^ Plus EdU Imaging Kits (Life Technologies). The images were obtained using an Olympus microscope. The analysis of immuno‐positive neurons in the hippocampal area was quantified with ImageJ software. Five brain sections were collected for quantifying the positive cells in each mouse.

### 2.5 Statistical analysis

The statistical analysis was performed using SPSS 16.0 software. Two‐way ANOVA, one‐way ANOVA, and Student's *t* test were performed for the data analysis. *p* < 0.05 was considered statistically significant.

## 3 RESULTS

### 3.1 EE improved the behavioral performance of APP/PS1 mice

**Figure 1:**
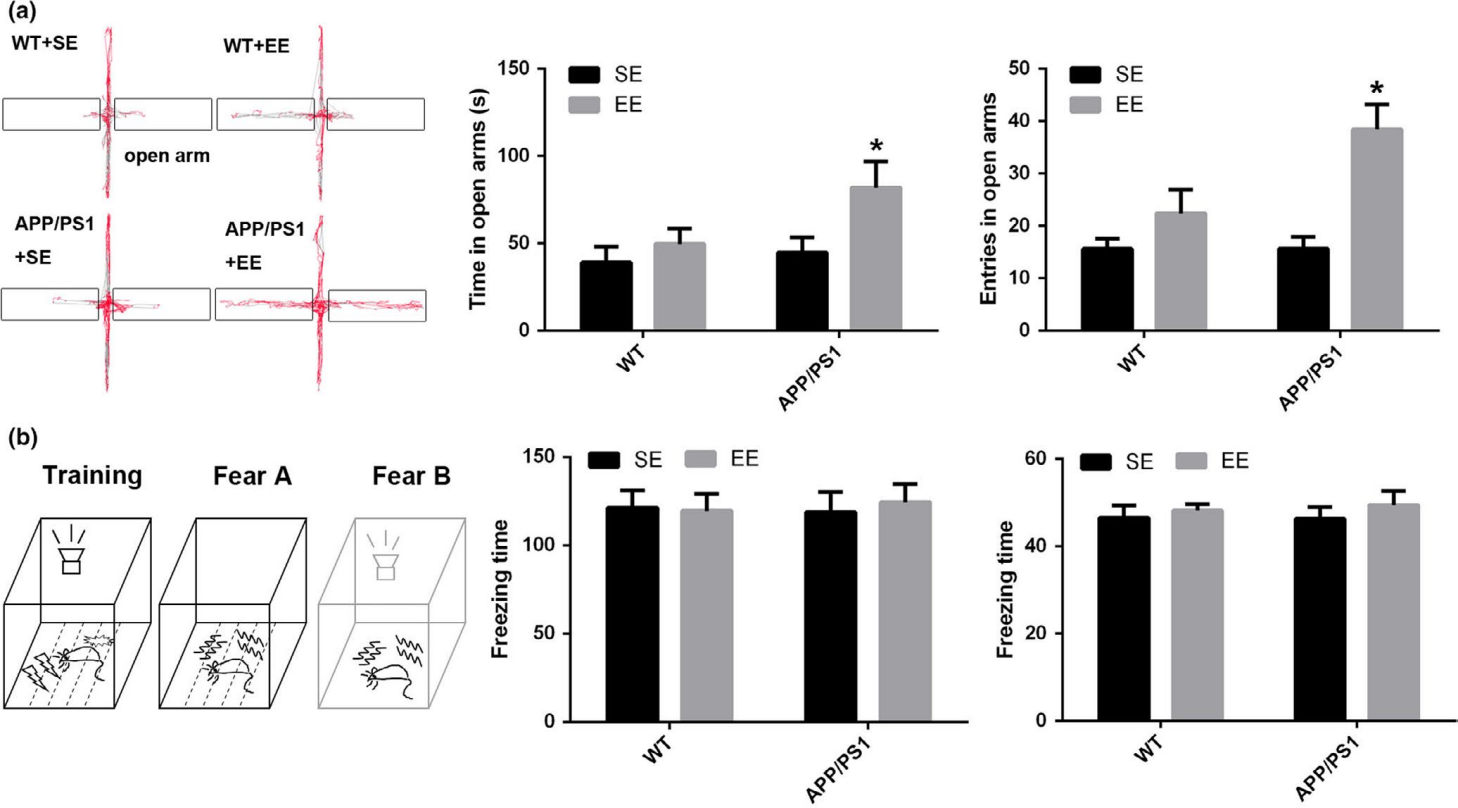
Elevated plus maze performance (a) Compared with the APP/PS1 mice that experienced SE, the time spent in and the frequency of entry into the open arms were both increased in the APP/PS1 mice that experienced EE. There was no difference in the WT mice after SE or EE. Fear conditioning performance (b) In the fear conditioning test, the freezing time in chambers A and B was not different among the four groups. WT + SE and WT + EE (*N* = 8 each group), APP/PS1 + SE and APP/PS1 + EE (*N* = 9 each group). *p < 0.05 *VS* APP/PS1 (analysis by two‐way ANOVA and further tested by one‐way ANOVA and Student's *t* test)

After 1 month of SE exposure, the mice were tested in the EPM. The results showed that there were no differences between the APP/PS1 mice and WT mice in the frequency of entry into or the time spent in the open arms (time: WT + SE, 39.17 ± 9.00 s; APP/PS1 + SE, 44.82 ± 8.73 s; *p* = 0.718; frequency: WT + SE, 15.63 ± 1.97; APP/PS1 + SE, 15.67 ± 2.27; *p* = 0.994; Figure 1a). After 1 month of exposure to EE, the time spent in and the frequency of entry into the open arms were significantly increased in the APP/PS1 mice (time: APP/PS1 + SE, 44.82 ± 8.73 s; APP/PS1 + EE, 81.96 ± 15.02 s; *p* = 0.046; frequency: APP/PS1 + SE, 15.67 ± 2.27; APP/PS1 + EE, 38.44 ± 4.74; *p* = 0.004; Figure 1a), suggesting improved locomotor accompanied by less anxiety in the EE‐exposed APP/PS1 mice. However, the time spent in and the frequency of entry into the open arms did not significantly change between the SE‐ and EE‐exposed WT mice (time: WT + SE, 39.17 ± 9.00 s; WT + EE, 49.65 ± 8.73 s; *p* = 0.516; frequency: WT + SE, 15.63 ± 1.97; WT + EE, 22.38 ± 4.53; *p* = 0.212; Figure 1a).

The mice then performed a fear conditioning task. The mice were placed into the chamber, and the freezing times were measured in both the fear A and fear B stages. The results showed that there were no significant differences in the cognitive index between these groups (time: WT + SE, 46.58 ± 2.78 s; WT + EE, 48.23 ± 1.50 s; APP/PS1 + SE, 46.33 ± 2.71 s; APP/PS1 + EE, 49.46 ± 3.27 s; *p* = 0.821; frequency: WT + SE, 121.49 ± 9.52; WT + EE, 119.58 ± 9.65; APP/PS1 + SE, 118.77 ± 11.73; APP/PS1 + EE, 124.39 ± 10.40; *p* = 0.981; Figure 1b).

### 3.2 EE increased the number of c‐Fos‐positive neurons in the DG area but not in CA1 and CA3

**Figure 2:**
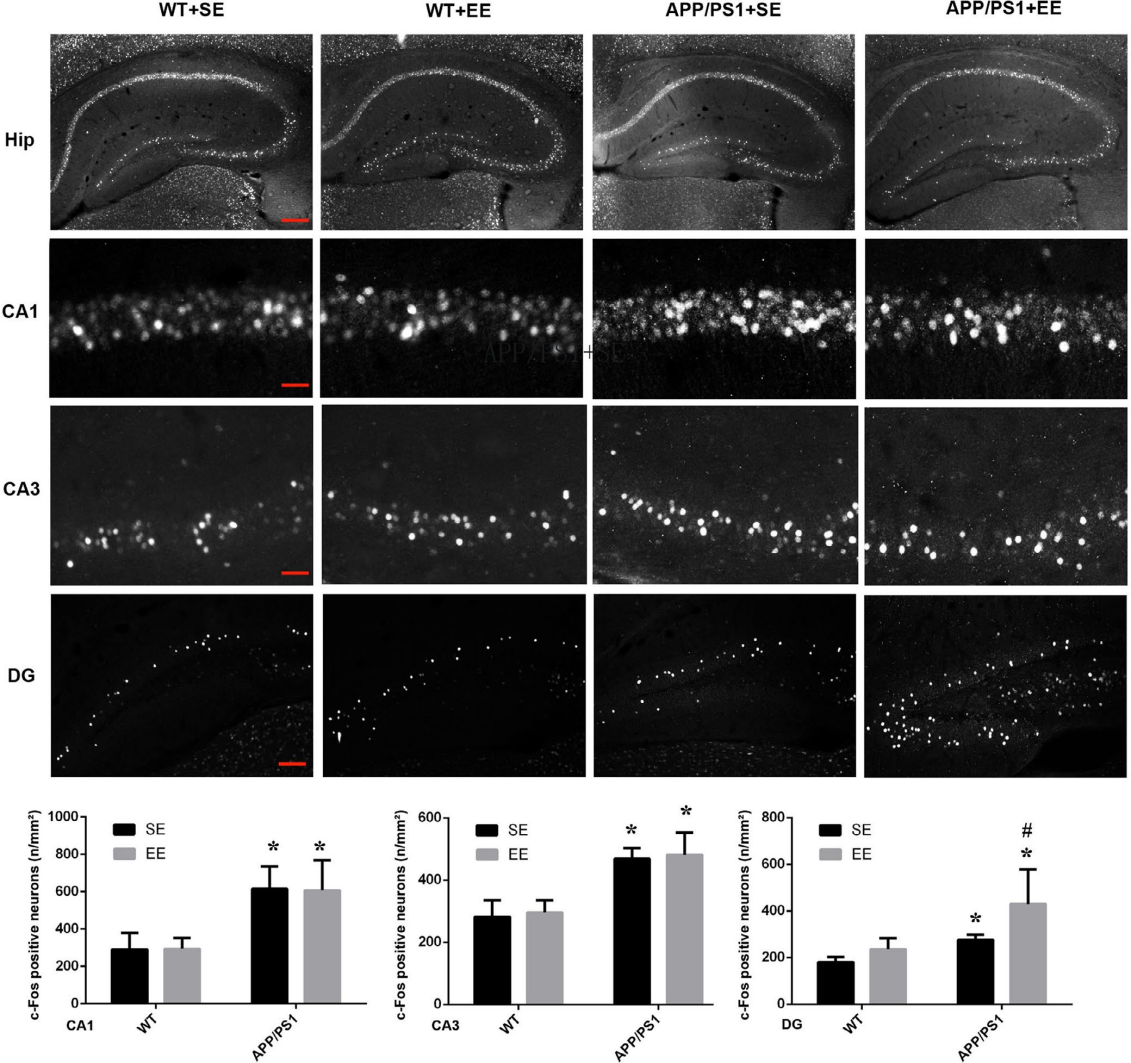
c‐Fos expression in the hippocampal area. In the CA1 and CA3 areas, compared with that in the WT mice, the expression of c‐Fos was increased in the APP/PS1 mice but was not altered after EE in both strains of mice. In the DG area, compared with that in the WT mice, c‐Fos expression was increased in the APP/PS1 mice, and EE further upregulated it significantly. The scale bar represents 200 μm in the Hip images, represents 20 μm in the CA1 and CA3 images, and represents 100 μm in the DG images. *N* = 3 mice in each group. *p < 0.05 *VS* WT, ^#^ *p* < 0.05 *VS* APP/PS1 (analysis by two‐way ANOVA and further tested by one‐way ANOVA and Student's *t* test)

The behavioral trials were then followed by the measurement of c‐Fos expression, which indicates the extent of neuronal activation, in the hippocampus. Consistent with the behavior performance on the EPM, a dramatic increase in the number of c‐Fos‐positive cells was observed in DG area of the EE‐exposed APP/PS1 mice (DG: WT + SE, 180.58 ± 12.27; WT + EE, 235.82 ± 27.56; APP/PS1 + SE, 285.77 ± 22.96; APP/PS1 + EE, 449.04 ± 105.03; Figure 2). However, in the brain regions of CA1 and CA3, an increase in c‐Fos‐positive neurons was observed in the APP/PS1 mice compared with the WT mice, and the number of c‐Fos‐positive neurons was not affected by EE exposure in either the WT or APP/PS1 mice (CA1: WT + SE, 289.80 ± 52.50; WT + EE, 294.16 ± 33.44; APP/PS1 + SE, 615.18 ± 68.81; APP/PS1 + EE, 607.05 ± 93.26; CA3: WT + SE, 281.98 ± 31.01; WT + EE, 295.23 ± 23.14; APP/PS1 + SE, 467.73 ± 20.55; APP/PS1 + EE, 480.92 ± 41.51; Figure 2).

### 3.3 EE increased hippocampal neurogenesis and activated c‐Fos expression in newborn cells in APP/PS1 mice

**Figure 3:**
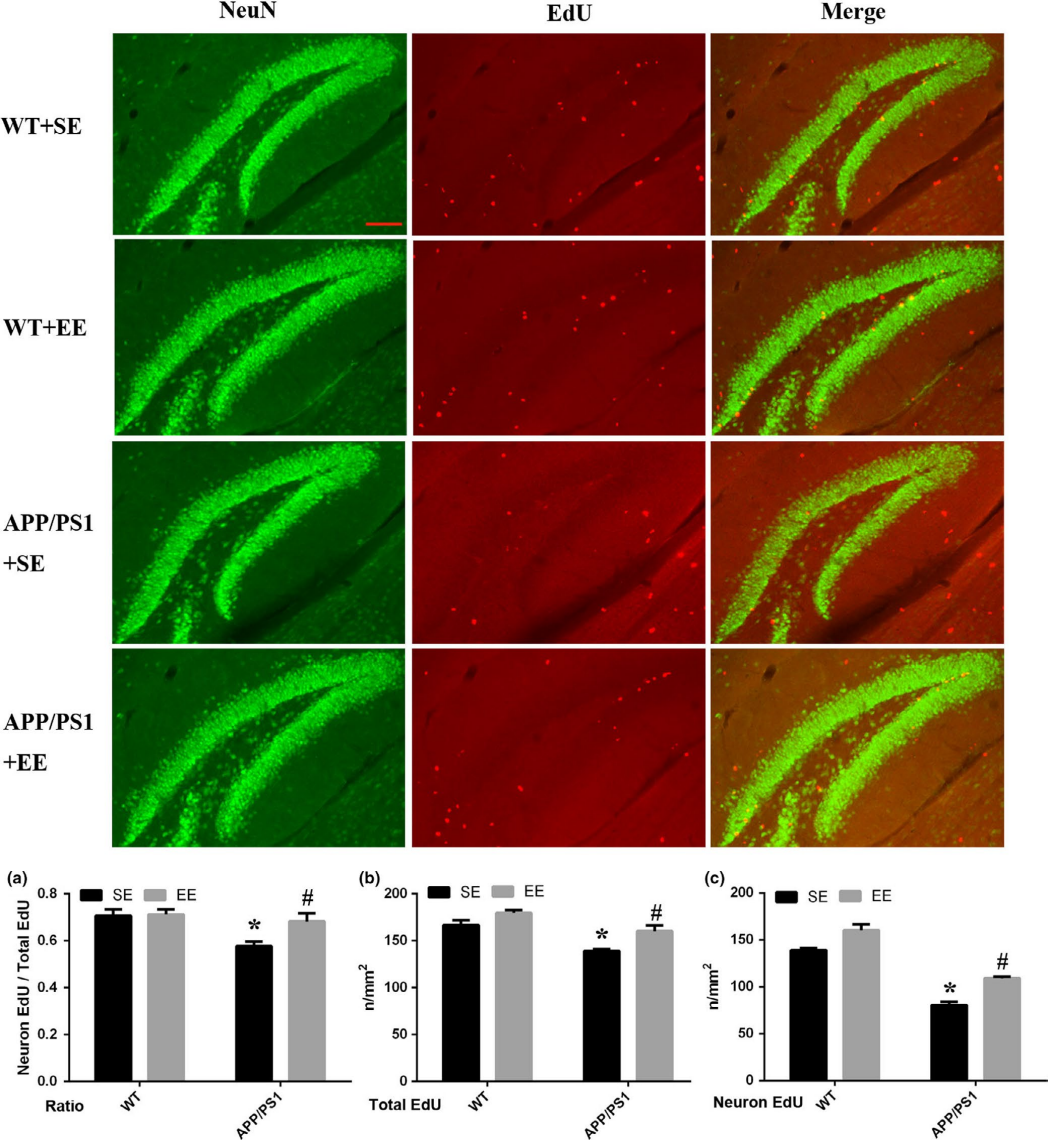
New‐born neurons in the DG area. Compared with those in the WT mice, neuronal EdU (C), total EdU (B), and ratio of neuron EdU to total EdU (A) were all decreased in the APP/PS1 mice, and EE rescued their expression significantly in the APP/PS1 mice. The scale bar represents 100 μm. *N* = 3 mice in each group. *p < 0.05 *VS* WT, ^#^ *p* < 0.05 *VS* APP/PS1 (analysis by two‐way ANOVA and further tested by one‐way ANOVA and Student's *t* test)

**Figure 4:**
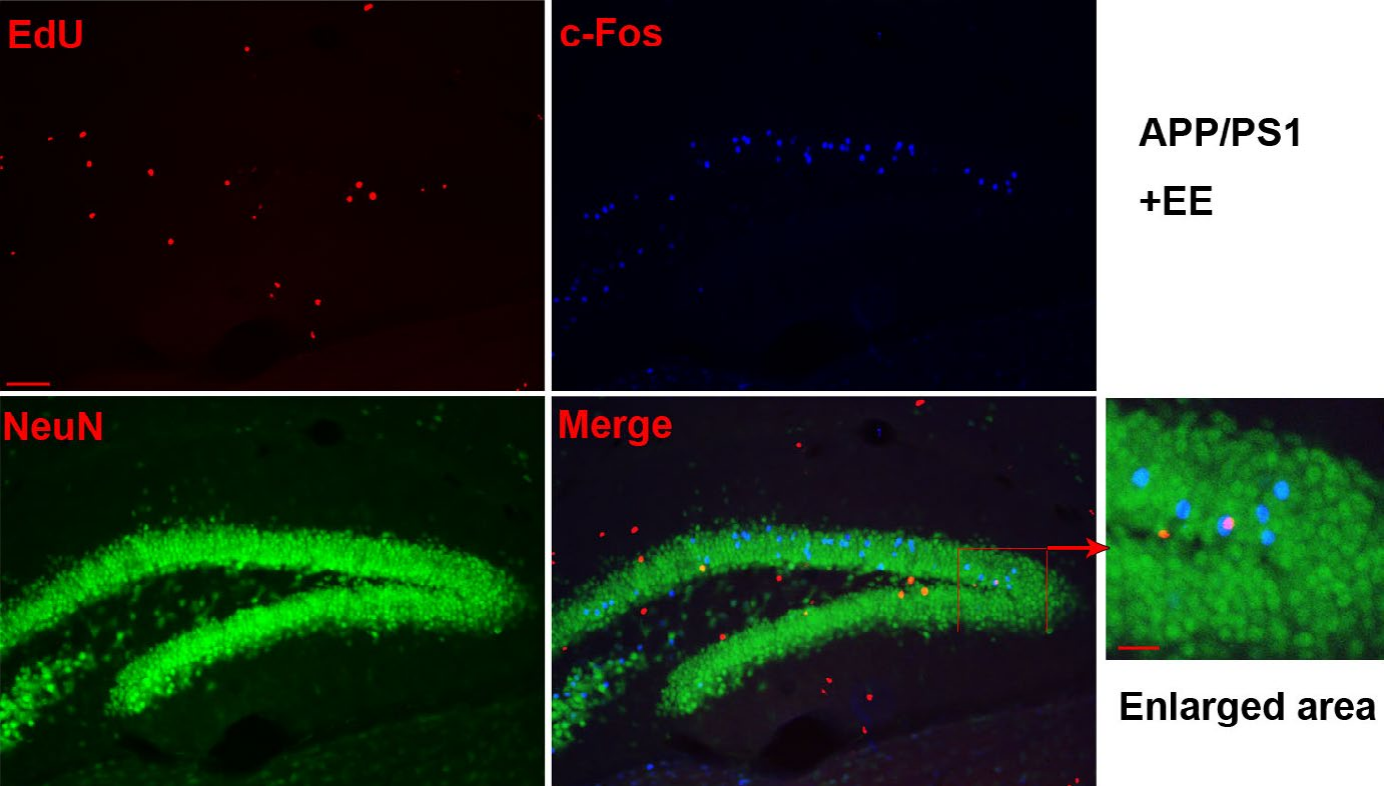
c‐Fos expression in newborn neurons in the DG area of the APP/PS1 mice after EE. These results showed that c‐Fos was expressed in newborn neurons in the APP/PS1 mice exposed to EE. The scale bar on the left represents 70 μm, and the scale bar on the right represents 25 μm

The results showed that, compared with the WT mice exposed to SE, the number of newborn neurons decreased in the APP/PS1 mice. However, that number was significantly increased in the APP/PS1 mice under EE conditions but not in the WT mice exposed to EE (neuronal EdU: WT + SE, 117.67 ± 0.67; WT + EE, 128.00 ± 4.04; APP/PS1 + SE, 80.33 ± 3.84; APP/PS1 + EE, 109.00 ± 1.73; total Edu: WT + SE, 166.67 ± 5.24; WT + EE, 179.67 ± 2.91; APP/PS1 + SE, 139.00 ± 2.08; APP/PS1 + EE, 160.33 ± 6.06; the ratio of neuronal EdU to total Edu: WT + SE, 0.71 ± 0.03; WT + EE, 0.71 ± 0.02; APP/PS1 + SE, 0.58 ± 0.02; APP/PS1 + EE, 0.68 ± 0.04; Figure 3). The results of c‐Fos, EdU, and NeuN staining indicated that in the EE‐exposed APP/PS1 mice, c‐Fos was expressed in newborn neurons (Figure 4), while there was no expression of c‐Fos in newborn neurons in the other three groups (Figure S1).

## 4 DISCUSSION

This study aimed to elucidate the effect of EE in the early stage of AD. There is evidence that the cognition of APP/PS1 mice is not damaged at this young age, and the changes observed in the fear conditioning task in the APP/PS1 mice in our study were consistent with a previous report (Webster, Bachstetter, Nelson, Schmitt, & Van Eldik, 2014). In contrast to many other studies that have reported improved cognitive function exhibited by AD mice exposed to EE (Arendash et al., 2004; Costa et al., 2007; Jankowsky et al., 2005), we were not able to detect such an improvement, which may be attributable to the unchanged baseline cognition at an early age in the APP/PS1 mice.

The alteration in motor performance indicated an effect of EE on the APP/PS1 mice. A motor phenotype in APP/PS1 mice has been previously reported (Wirths, Breyhan, Schafer, Roth, & Bayer, 2008), and it can be partially rescued by exposure to EE. APP/PS1 mice have abnormally lower levels of anxiety in comparison with those of WT mice, and this difference becomes significant at 2 months of age (Cotel et al., 2012). In our study, the analysis of the performance of the APP/PS1 mice after EE exposure on the EPM task revealed a significantly increased time spent in and frequency of entry into the open arms, suggesting that increased locomotion and decreased anxiety were exhibited by the APP/PS1 mice after environmental interaction, which is consistent with a previous study (Gortz et al., 2008). Another study showed enhanced anxiety‐like behavior in the TgF344‐AD rat model that represents an early‐stage behavioral marker in the AD model (Pentkowski et al., 2018). Although the anxiety‐related results are controversial, they show that abnormal anxiety behavior in the early stage of AD may be an early sign of the disease. However, the benefits of EE on anxiety‐related behavior and the exact mechanism by which early emotional dysfunction is associated with later cognitive impairment in AD remain to be studied. There is no alteration in WT mice after EE exposure, mainly because, at a young age, the emotions and body functions of WT mice are normal and can help them adapt well to both internal and external environmental changes.

Decreased hippocampal neurogenesis has been previously described in mouse models of AD (Donovan et al., 2006; Herring et al., 2009; Rodriguez et al., 2008; Verret, Jankowsky, Xu, Borchelt, & Rampon, 2007). Several studies have examined the effects of physical activity and EE on neurogenesis in 5‐month‐old and older mice, that is, the medium and late period of AD in mouse models (Herring et al., 2009; Mirochnic, Wolf, Staufenbiel, & Kempermann, 2009; Wolf et al., 2006). The results of a previous study demonstrated that EE upregulates hippocampal neurogenesis in adult mice (Catlow et al., 2009). Equally important is the examination of the effect of EE on young AD mice when the brain lacks major pathological changes that occur later in life. Very little information is available on the status of neurogenesis at the early stage of AD in mice and whether EE can rescue neurogenic impairments at this stage (Hu et al., 2010). In our study, we found that hippocampal neurogenesis was also impaired at a very early stage in APP/PS1 mice and could be rescued by exposure to EE.

Impairments in the survival and normal function of newborn cells, which were observed in the hippocampus of old APP/PS1 mice, suggest that the loss of newborn neurons in APP/PS1 mice occurs relatively late in maturation (Goncalves et al., 2016). Due to the higher excitability of newborn neurons, they are likely to have a significant impact on DG activity, despite their relatively low numbers (Goncalves et al., 2016). We also found an elevation in the excitability of newborn neurons, which was indicated by c‐Fos staining in the hippocampus of the APP/PS1 mice, especially in the DG area, and neuronal excitability was further increased after exposure to EE. We also found that, compared with that of the WT mice, the hippocampus of the APP/PS1 mice expressed more c‐Fos, which indicated elevated neuronal excitability in the AD mice. In addition, this finding has already been tested by electrophysiology techniques in our previous study (Wang et al., 2017). However, the exact mechanism underlying this unregulated excitability remains to be explored.

In conclusion, the APP/PS1 mice exhibited higher neuronal excitability and an impairment in hippocampal neurogenesis at the early stage, and these changes were accompanied by increased locomotion and lower anxiety. However, EE exposure increased neurogenesis and elevated the excitability of newborn neurons in the hippocampus, indicating that the increased neurogenesis and activation of newborn neurons may participate in the alteration of behavioral performance.

## CONFLICT OF INTEREST

The authors have no conflicts of interest to declare.

## Data Availability

The data that support the findings of this study are available from the corresponding author upon reasonable request.
