# Supplementary figures and images for: The activated newborn neurons participate in enriched environment induced improvement of locomotor function in APP/PS1 mice
Source: Brain Behav. 2019 May 15;9(7):e01316. doi: 10.1002/brb3.1316 (PMC6625533; doi:10.1002/brb3.1316)

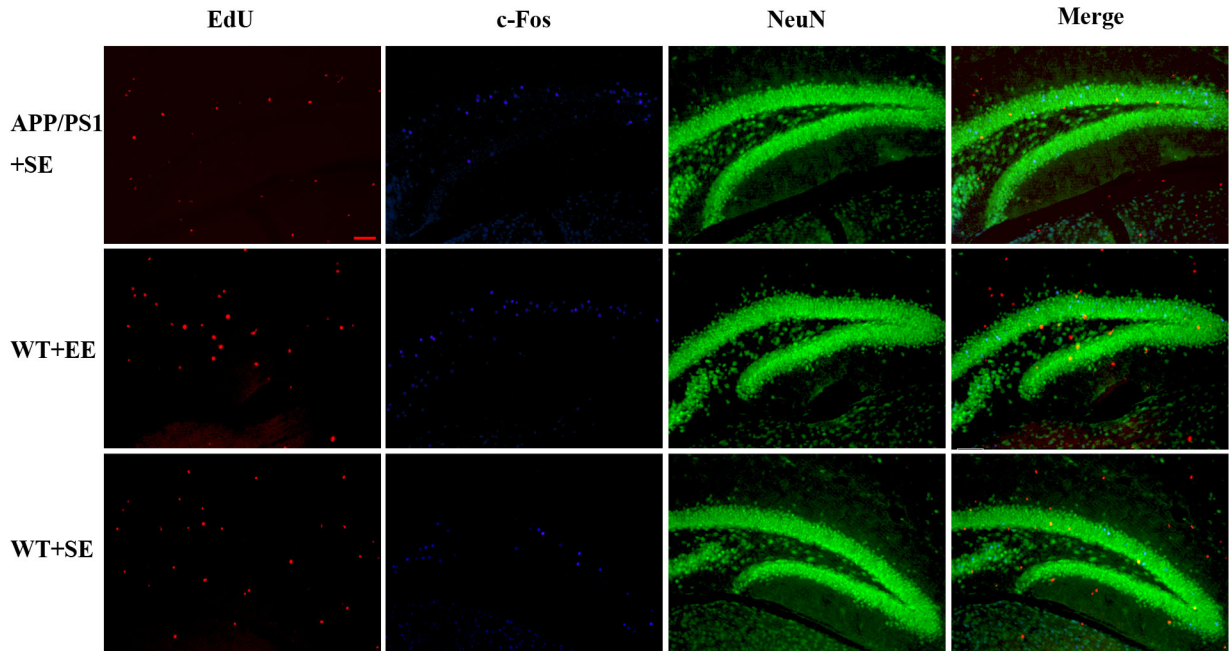

**Supplemental Figure 1**

Supplement: Supplementary file 1 [file BRB3-9-e01316-s001.pdf]
